# Potential Applications of Nano Silver Fluoride in the Prevention of Dental Caries in Older Adults: A Scoping Review

**DOI:** 10.1111/ger.70052

**Published:** 2026-02-02

**Authors:** Joel Acevedo Pico, Victor Gil Manich, Ruth Calleja Agudo, Gloria de Jaureguizar Marqués, Belisa Olmos

**Affiliations:** ^1^ Gerodontology, Special Care Dentistry and Oral Medicine at International University of Catalonia Barcelona Spain; ^2^ Special Care Dentistry and Oral Medicine at International University of Catalonia Barcelona and Sant Cugat del Vallès Spain

**Keywords:** dental caries, minimally invasive dentistry, Nano Silver Fluoride, older adults, scoping review

## Abstract

**Background:**

Dental caries remains highly prevalent in older adults and represents a growing public health concern due to increased tooth retention, polypharmacy, hyposalivation and functional limitations. Silver diamine fluoride (SDF) is widely used for caries arrest in this population but causes undesirable tooth staining. Nano Silver Fluoride (NSF) has emerged as a potential alternative, combining antimicrobial and remineralizing properties with improved aesthetics. However, its applicability in geriatric dentistry remains unclear.

**Objective:**

This scoping review aimed to identify and map existing evidence on the use of Nano Silver Fluoride for the prevention or management of dental caries in older adults.

**Methods:**

The review followed the PRISMA‐ScR guidelines framework. A systematic search was conducted in PubMed, Scopus, Web of Science, Cochrane Library, and SciELO for articles published between January 2020 and December 2024. Studies were eligible if they involved human participants aged ≥ 60 years, evaluated NSF for caries prevention or treatment and were published in English, Spanish or Portuguese. In vitro studies, animal models, case reports, reviews, and studies on children or young adults only were excluded.

**Results:**

A total of 157 records were identified; 14 full‐text articles were assessed. None met the inclusion criteria, as all clinical studies involving NSF were conducted in paediatric or young adult populations, lacked older adult participants, or were laboratory‐based. No clinical trials, observational studies, or reviews evaluating NSF in older adults were found.

**Discussion:**

This scoping review reveals a complete absence of clinical evidence regarding the use of Nano Silver Fluoride in adults aged 60 years or older. Although NSF has shown promising results in younger populations and is chemically related to SDF, its effectiveness and safety in geriatric dentistry remain unknown. High‐quality clinical studies in older adults are urgently needed.

## 1 Introduction

Dental caries remains one of the most prevalent chronic diseases globally and continues to pose a major public health challenge in older adults [1, 2, 3, 4]. Increased life expectancy has resulted in a higher number of older individuals retaining natural teeth, making them more susceptible to coronal and root caries [2, 5, 6]. Age‐related factors such as reduced salivary flow, polypharmacy, cognitive impairment, functional dependence, and limited access to dental care further contribute to the high incidence and progression of dental caries in this population [2, 6, 7]. Preventive strategies tailored to this age group are, therefore, essential.

Silver diamine fluoride (SDF) has been widely used as a minimally invasive and cost‐effective strategy for arresting caries without requiring operative intervention [8, 9, 10, 11, 12, 13, 14, 15, 16, 17]. However, its use is often limited by undesirable effects including dark staining of carious lesions, metallic taste, mucosal irritation, and potential adverse impact on bonding to restorative materials [17, 18, 19, 20]. These drawbacks have encouraged the exploration of alternative agents with similar or enhanced anticariogenic properties but fewer side effects [16, 21, 22, 23, 24, 25, 26, 27, 28].

Nano Silver Fluoride (NSF) has emerged as a promising alternative [4, 5, 6, 7, 8, 9, 10, 11, 12, 13, 14, 15, 16, 17]. It is composed of silver nanoparticles, chitosan, and sodium fluoride, combining antibacterial activity with remineralising potential [16, 18, 29, 30, 31, 32, 33, 34, 35]. Preclinical studies have demonstrated its strong antimicrobial action against cariogenic bacteria, biocompatibility, and ability to prevent demineralization without causing discoloration [16, 19, 20, 21, 22, 23, 24, 25, 26, 27, 28, 29, 30]. Clinical trials in children and young adults have further shown that NSF can arrest or prevent dentine caries, reduce cariogenic bacterial load, and is well accepted by patients due to its aesthetic advantages [27, 28, 29, 30, 31, 32, 33, 34, 35, 36, 37].

Despite promising outcomes in younger populations, evidence on the use of NSF in older adults remains unclear [38, 39, 40, 41, 42, 43, 44, 45]. No published reviews have synthesised available literature on its use in this age group [46, 47, 48, 49, 50, 51, 52, 53]. Given the clinical importance of caries prevention in older populations and the lack of synthesised evidence, a broader exploratory approach is necessary to map current research, identify existing knowledge gaps, and determine whether any clinical trials exist involving older adults [54, 55, 56, 57, 58, 59, 60, 61].

Accordingly, this study aimed to conduct a scoping review to identify and map the available evidence regarding the use of Nano Silver Fluoride for the prevention or management of dental caries in older adults.

## 2 Methods

This scoping review was conducted in accordance with the methodological framework. It also followed the Preferred Reporting Items for Systematic Reviews and Meta‐Analyses extension for Scoping Reviews (PRISMA‐ScR) guidelines [62]. The purpose of this approach was to systematically map the existing literature on Nano Silver Fluoride (NSF) in older adults, to clarify the extent and nature of the available evidence, and to identify gaps that require further investigation.

### 2.1 Research Question

The research question was developed using the PCC (Population, Concept, Context) framework. The population included older adults aged 60 years or above, according to WHO criteria. The concept focused on the use of Nano Silver Fluoride for the prevention or management of dental caries. The context included any dental care setting, regardless of geographic location, provided that the study involved clinical application or evaluation of NSF in humans. The research question was formulated as follows:

*What is the effectiveness of Nano Silver Fluoride (NSF) in preventing dental caries in older adults, according to literature published in the last five years?*

### 2.2 Search Strategy and Information Sources

A comprehensive literature search was carried out in the following databases: PubMed/MEDLINE, Scopus, Web of Science, Cochrane Library, and SciELO. Searches were conducted for studies published between January 2020 and December 2024. Grey literature sources, dissertations, and conference abstracts were not included. The search strategy combined Medical Subject Headings (MeSH) and free‐text terms such as ‘Nano Silver Fluoride’, ‘nanoparticle fluoride’, ‘dental caries’ and ‘older adults’. Searches in Spanish and Portuguese using equivalent DeCS terms were also conducted.

### 2.3 Eligibility Criteria

Studies were considered eligible if they met the following criteria: full‐text articles published in English, Spanish, or Portuguese; clinical studies in humans; use of Nano Silver Fluoride as an intervention for caries prevention or management; and inclusion of participants aged 60 years or older, or studies in mixed populations where data for older adults could be extracted. Studies were excluded if they were conducted in children or young adults only, were in vitro or animal studies, laboratory analyses, narrative reviews, systematic reviews, case reports, editorials, letters to the editor, or studies published before 2020.

### 2.4 Study Selection

All records retrieved from the databases were imported into a reference management software to identify and remove duplicates. Two reviewers independently screened titles and abstracts to determine relevance according to the eligibility criteria. Full texts of potentially eligible studies were assessed in detail. Discrepancies were resolved through discussion with a third reviewer. The study selection process was documented using a PRISMA‐ScR flowchart.

### 2.5 Data Extraction and Charting

A standardised data extraction form was developed using Microsoft Excel. From each included study, the following data were charted: authors, year of publication, country, study design, sample size, age of participants, description of the intervention, comparator (if any), follow‐up period, clinical outcomes related to caries prevention or arrest, and key findings. Due to the expected heterogeneity and absence of randomised trials in older adults, meta‐analysis was not planned.

### 2.6 Data Synthesis

Extracted data were synthesised descriptively. Results were narratively summarised to map the extent of research on NSF in older adults, identify the types of study designs available, summarise interventions and outcomes measured, and determine gaps in current evidence. No assessment of risk of bias or methodological quality was conducted, as this is not mandatory in scoping reviews and no eligible clinical trials were identified.

## 3 Results

**FIGURE 1:**
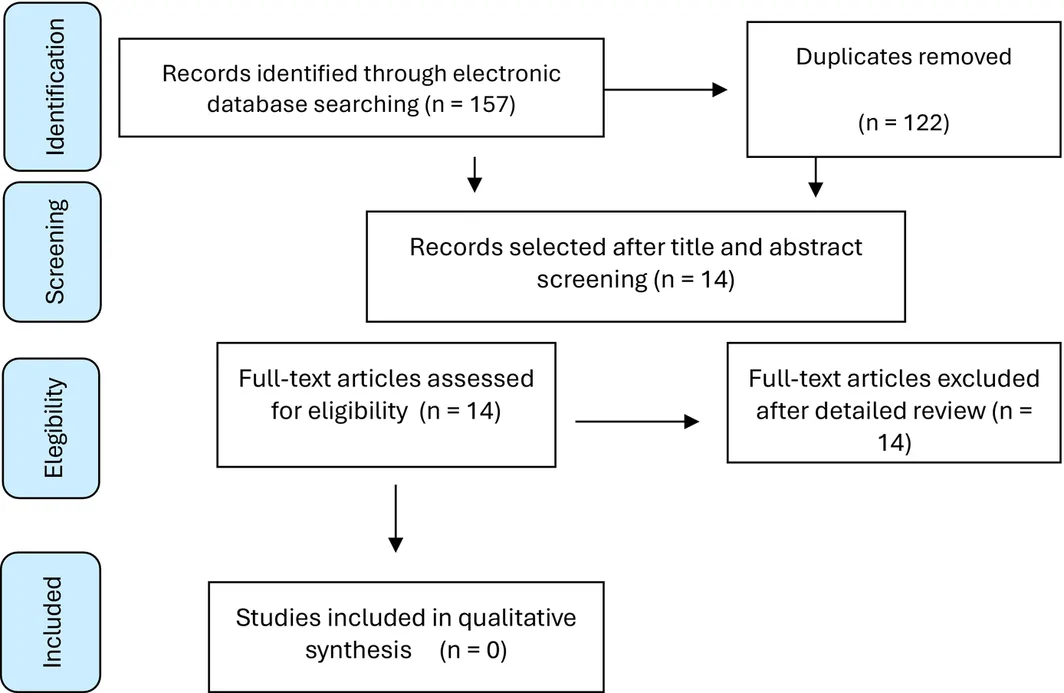
PRISMA‐ScR flow diagram of the study selection process. [Colour figure can be viewed at wileyonlinelibrary.com]

A total of 157 records were identified through the database search (Figure 1). After removing duplicates, 33 unique articles remained and were screened by title and abstract. Fourteen full‐text articles were retrieved for detailed evaluation. However, none of these met the eligibility criteria for inclusion in this scoping review. The study selection process is presented in the PRISMA‐ScR flow diagram.

The fourteen full‐text articles evaluated in detail primarily consisted of randomised clinical trials and quasi‐experimental studies conducted in children, adolescents, or young adults. These studies investigated the use of Nano Silver Fluoride to arrest active dentine caries, reduce cariogenic bacterial counts, or promote remineralisation of enamel or dentine. Some trials compared NSF with silver diamine fluoride, sodium fluoride varnish, or chlorhexidine, and overall reported promising antibacterial and remineralising effects, as well as favourable aesthetic outcomes. Nevertheless, none of the studies included participants aged 60 years or older, and none provided extractable data specifically related to older adults. Furthermore, certain studies were laboratory‐based, conducted in vitro or in animal models, and therefore did not meet the criteria of clinical human research. In some cases, NSF was assessed only as a surface pre‐treatment rather than as a caries‐preventive or therapeutic agent in clinical settings. Other studies lacked sufficient follow‐up time to evaluate outcomes relevant to caries prevention in older adults.

As a result, no clinical trials, observational studies, or reviews were found that evaluated the use of Nano Silver Fluoride in the prevention or management of dental caries in older adults. In addition, no previous scoping or systematic reviews addressing NSF in any population were identified. Due to the absence of eligible studies, no quantitative synthesis, risk of bias assessment, or subgroup analysis could be performed.

Overall, the results of this scoping review indicate a complete lack of clinical evidence regarding the application of Nano Silver Fluoride in older adults. While existing studies suggest potential benefits of NSF in younger populations, the current literature does not provide data to support its clinical use in geriatric dentistry. This highlights a clear gap in research and emphasizes the need for well‐designed clinical studies in older adult populations.

## 4 Discussion

Dental caries continues to represent a major public health concern in older adults due to increased tooth retention, hyposalivation, medication use and functional limitations that compromise oral hygiene [1, 2, 3, 4]. While silver diamine fluoride (SDF) has been widely studied and is considered an effective, minimally invasive option for caries arrest in frail or dependent elders [8, 9, 10, 11, 12], its clinical use is often limited by undesirable effects such as black staining, mucosal irritation, metallic taste and potential interference with adhesive procedures [12, 13, 14, 15].

These limitations have prompted the development of alternative materials, including Nano Silver Fluoride (NSF), which aims to maintain the antimicrobial and remineralising capacity of SDF while reducing aesthetic drawbacks [4, 16, 17, 18]. NSF is composed of silver nanoparticles, chitosan, and sodium fluoride, which together enhance antibacterial action, substantivity and remineralization [16, 19, 20, 21, 22, 23, 24, 25, 26, 27, 28, 29, 30, 31]. Several clinical studies in children and young adults have demonstrated that NSF can arrest dentine caries, reduce *Streptococcus mutans* counts and avoid the dark discolouration commonly associated with SDF [27, 28, 29, 30, 31, 61, 62, 63, 64, 65, 66, 67, 68, 69, 70, 71, 72, 73]. Despite these promising outcomes, all available clinical research has been conducted in younger populations, and no trial, observational study, or review has evaluated NSF in adults aged 60 years or older. The present scoping review confirms that evidence regarding NSF in geriatric patients is non‐existent.

In contrast, SDF has been extensively documented in older adults and is recognised as a reliable strategy for root caries management and non‐invasive caries control in patients with limited access to conventional dental care [8, 9, 10, 11, 74, 75, 76, 77, 78, 79, 80, 81]. The chemical and functional similarity between SDF and NSF suggests that NSF could be a potential alternative with improved aesthetic acceptance. However, biological conditions in older adults differ substantially from those of younger individuals, particularly in terms of saliva composition, polypharmacy, systemic diseases, prosthetic rehabilitation, and caregiving dependence [2, 3, 4, 5, 6]. Therefore, extrapolating results from paediatric or young adult populations to geriatric patients is not scientifically appropriate.

The absence of eligible studies found in this review indicates that NSF remains in an early developmental stage and has not yet been translated into geriatric clinical practice. Furthermore, no previous scoping or systematic reviews addressing NSF in any population were identified, highlighting how recent this topic is within cariology research. Given the growing interest in minimally invasive strategies for aging populations, future studies should evaluate NSF in older adults using robust clinical designs, adequate sample sizes, long‐term follow‐up and standardised outcomes such as root caries arrest, tooth sensitivity, patient‐reported perceptions and adverse effects.

In summary, although Nano Silver Fluoride has demonstrated antimicrobial and remineralising effects in younger populations, its application in geriatric dentistry has not been investigated. Until clinical evidence becomes available, SDF remains the most validated and clinically accepted option for caries control in older adults. Further research is essential to determine whether NSF can be a viable and aesthetic alternative for managing dental caries in ageing populations.

The complete absence of research on NSF in individuals aged 60 years and older highlights a significant gap in the literature. Future studies should therefore focus on evaluating the effectiveness, safety, patient acceptance and clinical outcomes of NSF in older adults, using standardised methodologies and adequate follow‐up periods. Until such evidence becomes available, the use of NSF in geriatric dentistry cannot be recommended and SDF should remain the preferred minimally invasive treatment for caries management in this population.

## Author Contributions

Joel Acevedo Pico: contributed to the conceptualization and design of the study, methodology, search strategy, data curation, study selection, data extraction, formal analysis, writing of the original draft, and project administration.

Víctor Gil Manich: contributed to supervision, validation, critical revision of the manuscript for important intellectual content, and writing, review, and edition.

Ruth Calleja Agudo: contributed to methodological support, interpretation of data, and writing, review, and edition.

Gloria de Jaureguizar Marqués: contributed to the interpretation of findings, manuscript refinement, and writing, review, and edition.

Belisa Olmos: contributed to academic oversight, supervision, and writing, review, and edition.

All authors have read and approved the final version of the manuscript and agree to be accountable for all aspects of the work, ensuring that questions related to the accuracy or integrity of any part of the work are appropriately investigated and resolved.

## Funding

This work was conducted independently and did not receive any specific grant from funding agencies in the public, commercial, or non‐profit sectors. The authors declare that the preparation of this systematic review was supported solely by institutional resources and the authors' own academic effort.

## Disclosure

Use of Artificial Intelligence: Artificial intelligence tools (ChatGPT, OpenAI) were used solely to support the improvement of English grammar, clarity, and structure of the manuscript. No part of the scientific content, data interpretation or conclusions was generated by artificial intelligence. All sources, facts and interpretations were developed and verified by the authors. The final version of the manuscript was thoroughly reviewed and approved by all authors to ensure accuracy and scientific integrity.

## Ethics Statement

This study is a systematic review of existing literature and did not involve the collection of new data from human participants or animals. Therefore, ethical approval from an institutional review board or ethics committee was not required. All included studies in this review were assumed to have obtained appropriate ethical approval and informed consent at the time of their original publication.

## Consent

The authors have nothing to report.

## Conflicts of Interest

The authors declare no conflicts of interest.

## Data Availability

The data that support the findings of this study are available from the corresponding author upon reasonable request.
